# Supplementary material for: A Cross-Species Analysis Reveals Dysthyroidism of the Ovaries as a Common Trait of Premature Ovarian Aging
Source: Int J Mol Sci. 2023 Feb 3;24(3):3054. doi: 10.3390/ijms24033054 (PMC9918015; doi:10.3390/ijms24033054)
Supplement: Supplementary file 1 [file ijms-24-03054-s001.zip › ijms-2113443-supplementary.pdf]

Supplementary Figures and Tables

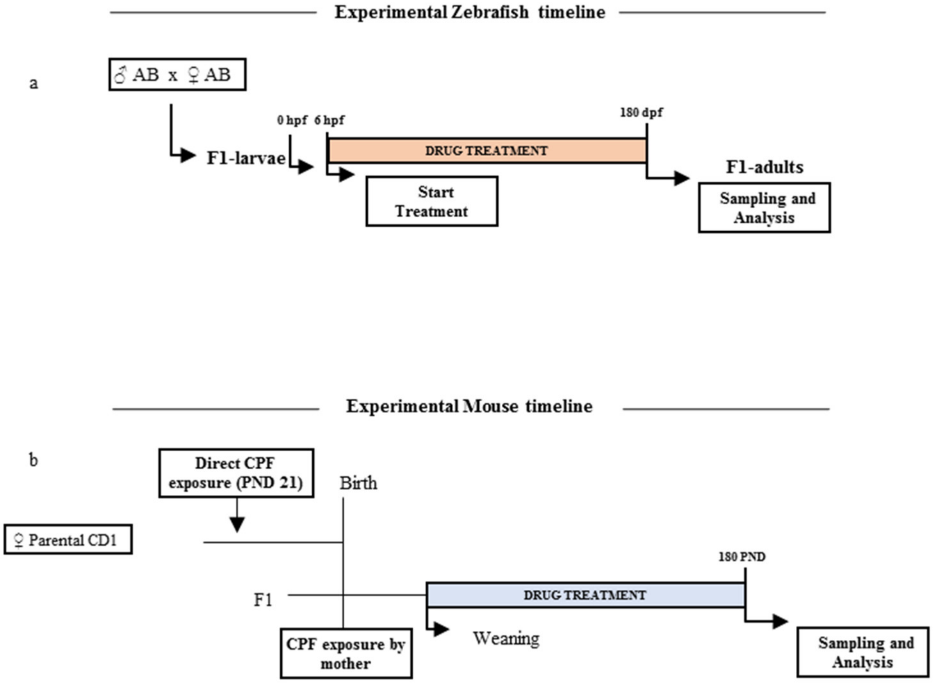

Figure S1. CPF experimental design in zebrafish and mouse models. (a) Treatment timeline in zebrafish animals. Embryos from wild type parents were collected and blastulae were selected. At 6 hours post fertilization (hpf) embryos were randomly assigned to experimental groups and treated until the adulthood (180 days). Detailed protocols are reported in M&M section. (b) Treatment timeline in mouse. Seven days before mating, female mice were exposed to CPF. The treatment of the mice enrolled in the studies was continued after the conception and at birth until the adulthood (180 days) by direct feeding. Details concerning mouse background and CPF concentration are described in Material and methods section (M&M section, from now on).

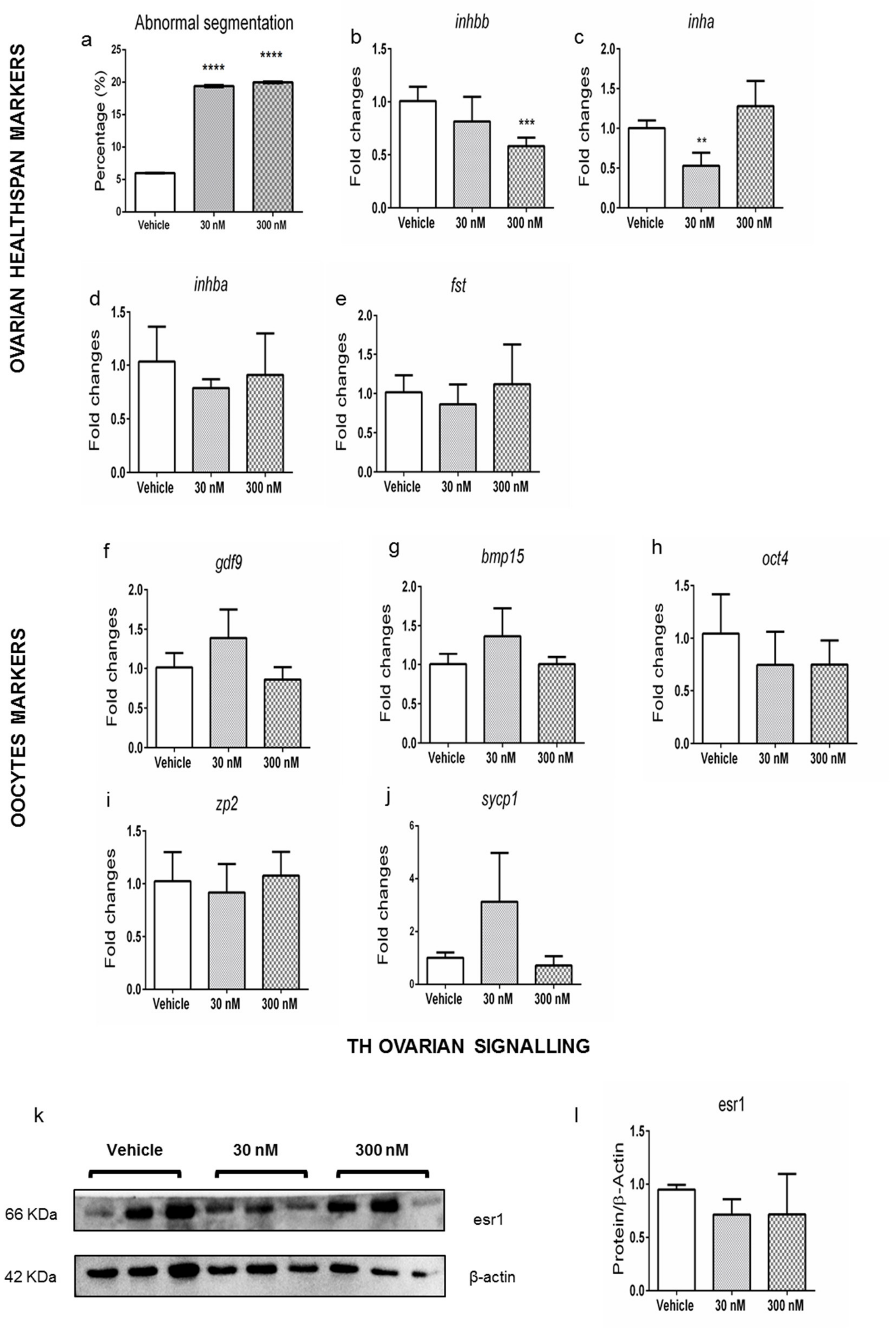

Figure S2. Developmental and lifelong exposure to CPF in zebrafish ovaries promotes POA. (a) Abnormal segmentation in zebrafish ovaries (b-j) Ovarian healthspan markers (*inhbb, inha, inhba, fst*) and oocytes markers (*gdf9, bmp15, oct4, zp2,sycp1)* were detected by RT-qPCR. (k,l) Representative Western blot analysis showing the level of esr1 protein following CPF treatment (N=3/group). Data were obtained normalizing using *tubaI* for mRNA and protein level. Data are mean ± s.d. with five animals for group. Significant differences are indicated with ** P < 0.01, *** P < 0.001 using Student's *t*-test.

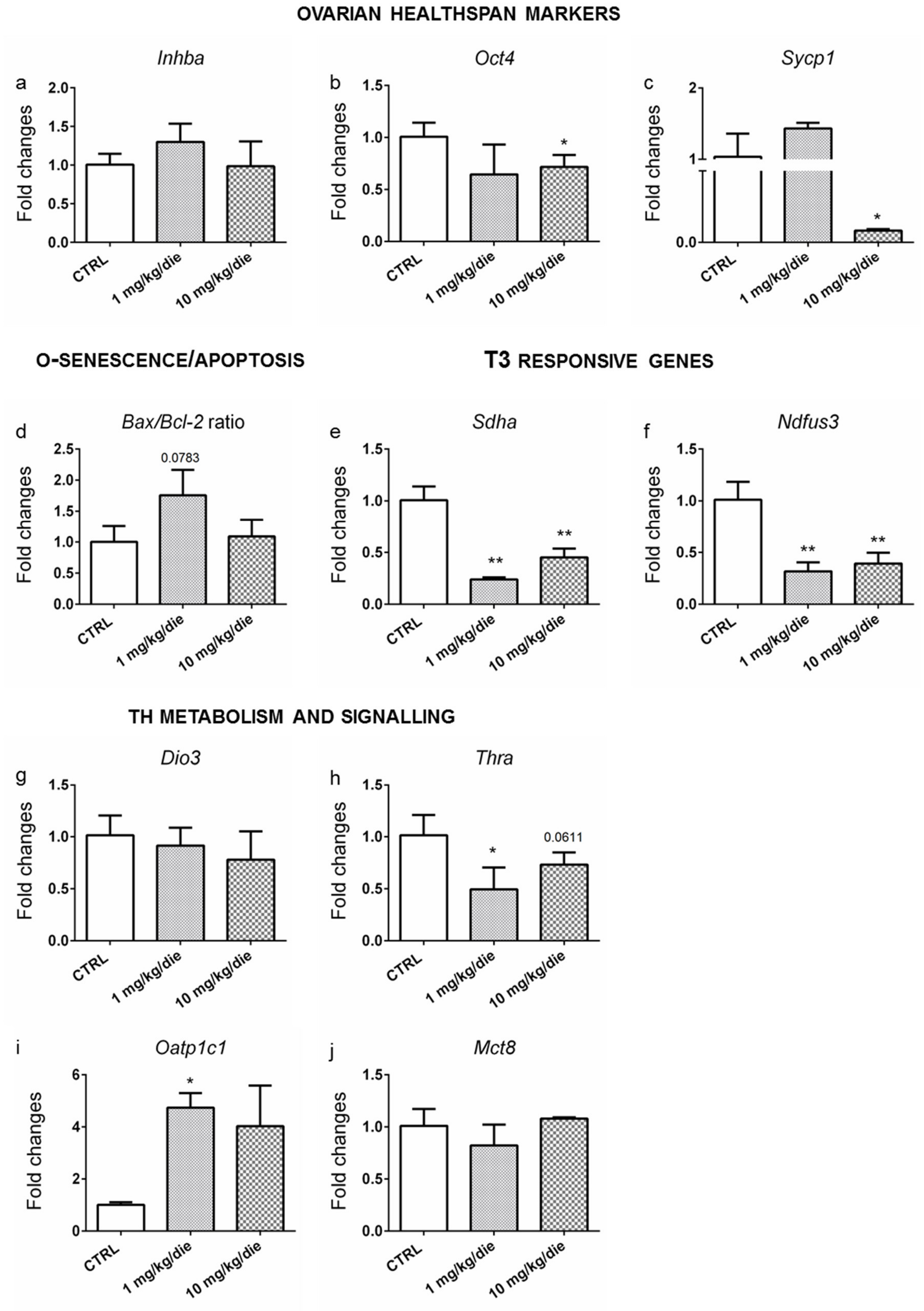

Figure S3. Developmental and lifelong exposure to CPF in mice ovaries promotes alteration in TH ovarian metabolism and signalling. (a-j) Ovarian healthspan markers (*Inhba, Oct4, Sycp1)*, *Bax*/*Bcl-2* ratio, T3 responsive genes (*Sdha, Ndfus3*) and genes implicated in TH metabolism and signalling (*Dio3, Thra, Oatp1c1, Mct8*) were detected by RT-qPCR. Data were obtained normalizing using *β-actin* for mRNA. Data are mean ± s.d. with five animals for group. Significant differences are indicated with *P < 0.05, ** P < 0.01, *** P < 0.001 using Student's *t*-test.

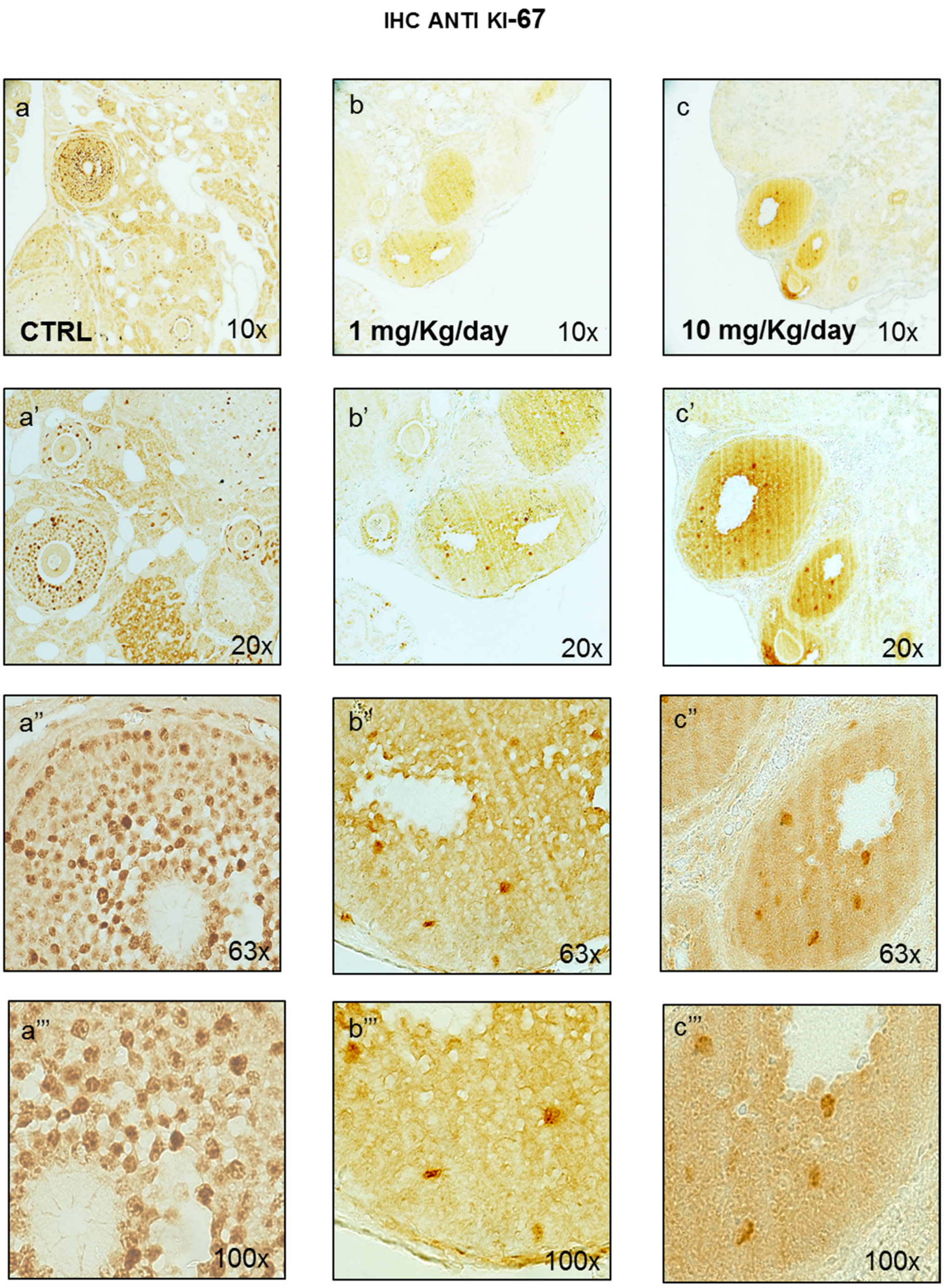

Figure S4. Developmental and lifelong exposure to CPF in mice ovaries reduce the GCs proliferations. (a-c''') Staining for Ki-67 on mice ovaries sections (10x, 20x, 63x,100x magnification), showing the alteration of Ki-67 level in exposed groups (1 and 10 mg/Kg/day) (N=3 ovaries/group).

Table S1. CT values of the genes implicated in TH metabolism and signalling. RT-qPCR analyses was presented and the CT means of each single biological replicate (N=5/group) are shown.

	CT values in Chow diets (mean)	
	C57BL/6J	FVB/NJ
Dio1	34.5	32.6
Dio2	26.6	26.1
Dio3	25.8	25.8
Thra	22.5	22.4
Thrb	22.2	22.2
Spot14	26.2	26.1

Table S2. Hormones active along the HPG-axis.

	Pituitary-Thyroid axis (*Tsh-β*, cfT3)		Pituitary-Thyroid-Ovary axis (cE2, *Fsh-β*, *Lh-β*)		
**Mouse exposed to CPF**	***Tsh-β*** (RT-qPCR	**cfT3** (ELISA)	**cE2** (ELISA)	***Fsh-β*** (RT-qPCR)	***Lh-β*** (RT-qPCR)
**CTRL**	1.0±0.2	2.9±0.4	22.0±8.8	1.0±0.1	1.0±0.1
**1 mg/Kg/day**	1.1±0.2	2.6±0.8	43.4±13.1 *	0.7±0.2	1.4±0.5
**10 mg/Kg/day**	2.0±0.5 *	1.8±0.7 *	15.1±6.9	0.2±0.05 **	1.2±0.2

The table reports the hormones active along the HPT-axis (*Tsh- β* and cfT3) and HPO-axis (cE2, *Fsh- β, Lh- β*) detected by ELISA assay and RT-qPCR. Data are mean ± s.d. with five animals for group. Significant differences are indicated with *P < 0.05, **P < 0.01 using Student's *t*-test.
